# The Doctrine of Signatures in Israel—Revision and Spatiotemporal Patterns

**DOI:** 10.3390/plants10071346

**Published:** 2021-07-01

**Authors:** Amots Dafni, Saleh Aqil Khatib, Guillermo Benítez

**Affiliations:** 1 Department of Evolutionary and Environmental Biology, University of Haifa, Haifa 3498838, Israel; amots.dafni@gmail.com; 2 Independent Resercher, Mghar 20128, Israel; salehaqil58@gmail.com; 3 Department of Botany, Campus Universitario de Cartuja, University of Granada, 18071 Granada, Spain

**Keywords:** medicinal plants, doctrine of similitude, Middle East, botany, history of plant uses, ethnobotany, medical anthropology

## Abstract

The present survey includes forty-three plant species with present-day medicinal applications that can be related to the Doctrine of Signatures (DoS). The main uses are for jaundice (33.3%), kidney stones (20%), and as an aphrodisiac (8%). Ten Doctrine of Signature uses (22.2%) are endemic (to Israel and Jordan); while none of these plant species are endemic to the region at all, their DoS uses are endemic. Summing up of all these data reveals that 73.2% of all uses found in present-day Israel could be considered as related to Muslim traditional medicine. About one quarter (24.4%) of the DoS uses are also common to Europe, and some (8.8%) to India. The two adventive species with DoS uses serve as evidence that the DoS practice is not necessarily based solely on its historical background but is still evolving locally in accordance with changes in the local flora. The current broad geographic distribution of many of the doctrine’s uses may serve as indirect evidence of its current prevalence, and not just as a vestigial presentation of ancient beliefs.

## 1. Introduction

The Doctrine of Signatures (henceforth DoS), or Doctrine of Similitude, claims that plants display characteristics, or “signatures”, such as color, shape, or common name, that are indicative of the disease that they can cure [1] (p. 210), [2] (p. 214). Throughout human medical history, people have used plants, minerals, and animals as medicines to treat diseases and body organs that are similar in their color, shape, or even name to the disease’s symptom. Tippo and Stern [3] (p. 470) state: “In many cases, a firm belief in the goodness of God who put everything on earth for his people gave rise to the doctrine of signatures, which held that the key to man’s use of plants was hidden in the form of the plant itself; one had only to look closely”.

Stretching back into antiquity, practices associated with the DoS were widespread, yet scholars have seldom offered a clear explanation for the origins of this pervasive medical philosophy. Some insist that the DoS originated in China [4] (p. 581) and spread throughout Europe during the Middle Ages [5] (p. 289), which seems unlikely, given the early mention of practices associated with the DoS in Ancient Rome (e.g., Dioscorides’s *Materia Medica* [6]. Some modern academics believe that the early Egyptians derived their medical knowledge from these signs [2] (p. 214). While some authors [5] (p. 289) [7,8,9] have suggested that the DoS is universal, since similar patterns of ‘resemblance’ have been observed in ancient Asia, classical Greece, medieval Europe, and pre-Columbian America, others [9] suggest it is a European idea, with a written origin.

The theory was first published by Theophrastus Bombast von Hohenheim (1493–1541), better known as Paracelsus, in his book *Dē rērum natūra* (Supreme Mysteries of Nature, 1656 (see: [10] (p. 250) and [11] (pp. 25–26))). This theory was adopted and developed by Giambattista Della Porta in his *Phytognomonica*, first published in Naples in 1588 [12]. Their example was followed by Jakob Boehme (1575–1624), who, at the age of 25, had a profound mystical vision in which he believed the true relationship between God and man had been revealed to him. As a result of his vision, he wrote *Signatura Rerum* (The Signature of All Things), which was initially published in the first half of the 17th century [13] (p. 32). The DoS was enthusiastically recommended in Britain by William Coles (1626–1662). In his *Art of Simpling*, Coles [14] (p. 88) defined it: “Though sin and Satan have plunged mankind into an Ocean of Infirmities, yet the mercy of God, which is over all His workes, maketh Grasse to grow upon the Mountains, and Herbs for the use of men, and hath not only stamped upon them a distract forme, but also given them particular Signatures, whereby a man may read, even in legible characters, the use of them”. Other important authors dealing with the topic from epistemological or historical points of view include Foucault [15] and Crollius [16]. Agamben [17] performed a detailed study on the theory as a paradigm.

The DoS is found in medicinal systems all over the world and prevails throughout the whole range of folk medicine [8,9,18,19,20,21,22,23,24]. The DoS is alluded to in classical Greek literature on medicinal plants (see examples in results); however, it is often dismissed as primitive superstition [20,21,23].

The DoS is occasionally referred to consciously by healers who acknowledge that a plant cures only through the belief in its form [20,25,26]. Etkin and Johns [18] suggest that the mnemotechnical character of signatures might be related in varying degrees to empirical experience with medicinal plants. Leonti et al. [26] assume that the DoS serves as a mnemonic aid, facilitating the remembering of plant uses and medicinal traditions. Pattanayak and Nakak [27] (p. 1592) claim explicitly: “the DoS is a value system of a community to remember the medical importance of plant and animal species”. According to Bennett [20,21], historical discussions of the DoS represent post-hoc interpretations by scholars who attempt to explain why a plant is used for a particular ailment. According to him, there is little evidence that a priori analyses of morphological signatures led to the discovery of any medicinal plant. He also argues that most of the signatures are post-hoc appellations rather than a priori clues. A more general view is expressed by Durant [9], who suggests that “it is now time we stopped viewing the DoS as a doctrine at all, but rather a series of interwoven, but distinct, ethnomedical concepts which, given a more sympathetic interpretation, could pass the insight of previous generations into our hands”.

Our main objective is to find out whether the local population has any traditional knowledge about DoS plants, even if they do not know what this doctrine is, its origin and foundations. The aims of this study are: 1. To update the initial list of DoS plants in Israel [19] based on our additional field survey and the relevant recent literature. 2. To elucidate whether there are any spatiotemporal patterns of DoS uses by employing historical, phytogeographical, and ecological analysis as well as ethnobotanical data. 3. To survey modern pharmacological studies related to specific DoS uses of these plants in order to see if there is any possible relationship between DoS-specific uses and modern science.

## 2. Results

### Plant Species Used According to DoS

The list of plants used according to the DoS is presented in Table 1.

The present survey shows that 43 plant species have present-day DoS uses (Table 1). As two of them have two independent uses, we gathered information on 45 DoS uses for the study area. The main uses are for jaundice (33.3%), kidney stones (20%), and as an aphrodisiac (8%).

Most of the DoS uses are related to the Muslim medicine (73.2%). Among them, 24.4% of uses are also common in Europe, and 8.8% in India.

## 3. Discussion

### 3.1 DoS Categories of Use

The Holy Land (Israel and Palestine) is an area in which, throughout its long history, many cultures have amalgamated through the generations. The coexistence of different ethnic groups of various origins enables the absorption and the maintenance of old traditions, even in a single village. Thus, it is not surprising that there are still active remnants of old practices, including the DoS [20].

The medicinal DoS uses of plants in Israel can be divided into four categories. Each category expresses a plant’s specific external characteristic, indicating the disease for which the plant is used as a remedy or cure. The four categories, which were based on our data and the common concepts of the DoS in the literature [20,165], [174] (p. 16), are as follows:

#### 3.1.1. Similarity of Plant or Part of a Plant to a Damaged Human Organ

Nine plant species with kidney-shaped seeds are used in the treatment of kidney disorders (Table 1). Except for *Cynoglossum creticum*, all are legumes.

The roots of *Mandragora autumnalis* (mandrake), which resemble the human body, have been used to cure impotence and as an aphrodisiac since ancient times. Dioscorides [6] (I.570) already noted this similarity and use. Also, its fruits could be likened to testicles, explaining its specific use as an aphrodisiac throughout history. This tradition was highly evolved, especially during the Middle Ages [175,176,177], and is prevalent even nowadays [85]. The fruits of the mandrake are known as the most famous aphrodisiac in the history of the Western world since Biblical times (Genesis, 30: 14–16). This fruit resembles human testicles; hence, two of its Arabic names are “Goula’s (witch’s) eggs (testicles)” (بيض الجن) and “the Genie’s eggs (testicles)” (بيض الغول). Another Arabic name is حب التآلف, which means “the fruit that gets the lovers close”. Due to the fruit’s resemblance to a testicle and its use for fertility, this could be considered an additional example of the DoS.

The Greek term orchis (ὄρχις) was applied to the orchid plant by the botanist Theophrastus (~372–288 BCE [63] (9.18.3), since its root-tuber (and that of its related genera) has the shape of two testicles, which gave the name to the entire Orchidaceae family. He mentioned the medicinal use of orchids as aphrodisiacs and promoters of virility. Similarly, the fruits of *Astragalus macrocarpus* have a testicle shape, and hence its Arabic names mean testicles خْصيوِة) and fox’s testicles (خصّةالثعلب), and it is used as an aphrodisiac.

The flowering stem of *Cynomorium coccineum* looks like a human male sex organ. The meaning of the scientific name is “the red dog’s penis”. In Arabic, its name (زبّ الأرض) means “Earth’s penis”. This is, of course, the very reason for its use as an aphrodisiac.

The leaves of *Melissa officinalis*, which are shaped like a human heart, are used, according to Ibn Sina, as a medicine for heart weakness [77] (p. 189). One of our informants noted: “the leaves of the Melissa resemble a heart and a “tea” from it strengthens it” (**16**).

*Adiantum capillus-veneris* is a “classical” ancient example of the DoS (Table 1). The scientific name means “Venus’s unwettable hairs”. According to Greek mythology, Venus arose from the sea with dry hair [2] (p. 424). Theophrastus [63] (7.14.1) notes that the plant was used to treat the roots of hair.

#### 3.1.2. Similarity to Animal Shape or Behavior

Three species from our survey fit into this category. The inflorescence of *Heliotropium europaeum* resembles the abdomen of a scorpion; the plant has therefore been used to treat scorpion stings since the time of Dioscorides [6] (4.190). *H. europaeum* is called “scorpion plant” (*akrep out*) in Turkish [15] (p. 116). *H. arabinense* is the local vicariant as a desert replacement [178] (p. 865).

The compact, heart-shaped spikes of the species *Bryza maxima* resemble a tortoise. When the stalks shake in the wind, they resemble the courting behavior of the male tortoise pursuing a female; therefore, they are used in popular medicine for increasing love and affection [118] (p. 127). The idea is that the tortoise is persistent in his courtship; thus, a man who puts this plant under his mattress will acquire the same ability [19], [85] (pp. 112–113).

#### 3.1.3. Similarity of Plant Color to the Color of the Disease or Medical Phenomenon

Color similarity is one of the most common and well-known characteristics of the DoS. We found that 15 species which have yellow plant organs (mainly flowers or fruits) or yellow sap are most frequently used to treat jaundice. This fits with the findings of Patil [22], who states: “Yellow flowers, latex and dye are remedial to treat jaundice, a disease in which the patient is turned yellow”, and Suppan [4] (p. 581): “Traces of the belief are evident in the medicine of ancient India, where we find plants with yellow flowers recommended for the cure of jaundice”.

*Rhamnus alaternus* is called in Arabic “the yellow tree” (شجرة الصُّفيْر) in a reference to its yellow sap, which is used to treat jaundice. One of the names of *Ecballium elaterium* in North Africa is balḥat a-ṣufēr (بلحة الصُّفير), “the date’s fruit of the jaundice”; the fruit resembles dates, and the sap of its fruits is used to treat jaundice [179] (p. 528). This use was already mentioned by Ibn Sina [77] (p. 268) and is widespread all over the Mediterranean. A person who has jaundice is described in Arabic (**7**) as “his face is yellow like the flowers of *Glebionis”* (فلان وجهه أصفر مثل نوّار البسباس, *Glebionis coronarium*). The condition is completely correlated with this plant, which is used in Israel to treat jaundice.

*Rhus coriaria* has red fruits; this is the very reason why it was used to treat hemorrhage [165] (p. 17); the same is the case for red seeds in Mexico [26] (p. 31).

#### 3.1.4. Similarity to Plant Habitat or Characteristics

Several rock and wall (rupiculous) plants are believed to have the ability to dissolve stones (mainly kidney stones) in the same way that their roots penetrate the solid substrate in which they grow.

*Ceterach officinarum* has several vernacular names related to the kidney: one of its Italian names means “stone breaker” [153] (p. 8).

The fronds of *Adianthum capillus-veneris* shake with any slight movement of the air, so the plant is recommended against trembling because of fear [118] (p. 124), [104] (p. 2370). The leaves of *Ruta* spp. look like the palm of an open hand, which is a widespread omen to ward off the evil eye [180] (p. 227); they are very famous for this purpose around the Mediterranean [85] (pp. 56–58), [155] (passim).

*Verbascum* spp. has stellate hairs which may cause eye irritation and even temporary blindness on contact. One of our informants (**21**) told us that during the First World War, youngsters who wanted to escape the forced draft to the Turkish army used to rub their eyes with leaves of *Verbascum*. The inflammation developed was serious enough to release them from the army. One of its Arabic names means “the causer of blindness” ((**عَوَرْوَر**.

The color of the petals of some *Glaucium* species is red, which resembles the redness of inflamed eyes [165] (p. 19). This use is already mentioned by Ibn Baytar [45] (IV, p. 124–126). *Anastatica hierochuntica* has a hygrochastic mechanism of seed dispersal. The dried plant closes like a tightened fist in which the seeds are preserved until the rainy season. When the plant is wetted, it opens and the seeds are dispersed; this process may be repeated several times. In the Arabic folklore, the plant symbolizes the woman’s womb opening during birth; therefore, they wet the dried plant in a special magical ceremony ([38] (p. 131), [168] (pp. 122–123), [172]), to ease labor. 

### 3.2. Spatiotemporal Patterns

An analysis of the DoS uses shows several patterns concerning their spatiotemporal distribution, which are not mutually exclusive (Table 1). Regarding the time scale, for most species we cannot pinpoint when they were first recorded in a DoS usage or whether there is continuity of use since antiquity. But at least six uses (11.1%) are already mentioned in Ancient Greece/Rome and seven additional ones (15.5%) in the Middle Ages (Table 1).

Two species are adventive in the studied area; thus, their DoS use is surely new. *Oxalis pes-caprae*, native to the Cape region in South Africa, was first recorded in Israel in 1906 [181] (p. 9); we assume that it reached Jordan shortly after due to its rapid vegetative propagation. This species reached Libya in the second half of the 19th century (Figure 2; [182]). In both countries, it is used (independently?) against jaundice due to its very conspicuous yellow flowers. We were unable to find any record of this species having been used against jaundice elsewhere. In other countries, *Oxalis pes-caprae* has other medicinal uses: Syria [183] (p. 126) [184] (p. 125), Cyprus [110] (I, p. 61), and Turkey [84] (p. 146). *Nicotiana glauca*, native to South America, was first recorded in Israel in 1898 [181] (p. 49), and it is also logical to assume that, due to its aggressive spread, it invaded Jordan not too long thereafter. The use of this plant to treat jaundice is not known elsewhere [132] (p. 135). Among the Bedouins in Israel ([141] (p. 2119), [104] (105)), in Jordan [183] (p. 126), and in Cyprus [110] (I, p. 56), *N. glauca* has other medicinal uses that are not related to the DoS.

These two striking examples show that the DoS practice does not necessarily require an old traditional usage; the doctrine is still dynamically evolving in accordance with changes in the local flora. These cases seem to be parallel to the use of *Argemone mexicana* L., a plant of American origin [185], which has many medicinal uses outside its native area, including to treat jaundice in India ([186] (p. 2205), [187]), and also in Saudi Arabia [188] (p. 162) as a DoS plant. This species also has other medicinal uses in Jordan [183] (p. 126), Cyprus [110] (I, p. 56), Turkey [96] (p. 828), and Israel ([104] (p. 105), [141] (p. 2119)).

With regard to the spatial patterns, no fewer than ten DoS uses (22.2%) are endemic (Israel, Palestine, and Jordan are regarded as one phytogeographical unit). All these plant species are not endemic to Israel/Palestine and/or Jordan at all, *but* their DoS uses are. Four additional DoS uses are limited to the Middle East, and 10 more are also present in North Africa. Summing up of all these data shows that 73.2% of all the DoS uses related to present-day Israel could be considered as being related to Muslim traditional medicine, as could be inferred from their geographic spread in Muslim territories (Table 1).

Apart from endemic DoS uses, we should highlight the fact that two plants have the same DoS use in different and geographically distant territories (disjunct distribution of the use): *Oxalis pes-caprae* (Israel and Libya), and *Dittiricha viscosa* (Israel and Italy); both are used to treat jaundice. Geographical disjunction could be a result of three different factors: independent evolution of the use in both territories, the result of a previously wider distribution that shrank due to ecological changes and/or human activities, and cultural migration. In these two cases, we have no supporting data to sustain any specific possibility.

Four plants show vicarious distribution of DoS uses: *Glebionis segetum* in Turkey vs. *G. coronarium* in Israel to treat jaundice, and various *Orchis* spp. around the Middle East and Europe. In Israel, *Heliotropium arabinense* (which has a narrower distribution, growing exclusively in deserts) replaces *H. europaeum* (with a wider distribution); the same can be said for *Asphodelus tenuifololius*, which replaces *A. macrocarpus* in the desert, and *Verbascum eremobium*, which replaces *V. sinuatum* since these two pairs are ecological vicariants which replace each other in different vegetational territories. It seems that in the last three cases, we have a kind of cultural shift from the common type of a widely distributed species to a local one.

The large portion of endemic DoS uses reflects a deep-rooted local adaptation of a ubiquitous old theory. The incorporation of the adventive species is an indirect kind of evidence for an active process of continuing evolution of the DoS in local herbal medicine. Moreover, the current large distribution of many of the DoS uses recorded (Table 1) may serve as indirect evidence of its current prevalence and not just as a vestigial presentation of ancient beliefs. DoS uses are, therefore, deeply rooted in the culture of herbalism in Israel and Palestine but, instead of being a static store of knowledge, it is also open to innovation.

### 3.3. Theoretical Considerations

Efferth and Greten [23] consider the DoS valuable from a historical perspective as a means to describe and understand medieval medicine in Europe and elsewhere. They mention considerable scientific skepticism toward the idea that plant shapes and colors help in the discovery of medicinal uses of plants. However, once a medicinal use with curative effectiveness for a particular plant coincides with its shape or color, selective perception may promote the persistence of the DoS.

Leonti et al. [26] propose that the DoS is essential in the maintenance of medical traditions, but it cannot be studied as well as the humoral system in biomedical or bioscientific terms. However, the DoS species used may well be of interest with respect to ethnopharmacological effects on symptoms or illnesses treated with these remedies. Anyhow, the idea cannot be discarded because we cannot, for instance, deny the inexplicable role played in human health by culture, belief, psychology, and the “placebo effect” [189] (p. 110).

Bennett [20] investigates whether plants with heart-shaped leaves were used in cardiac medicine. Out of 80 species randomly selected from a literature review, 21 were used as medicines, and only three were used in cardiac medicines. He concludes that there is no support for the DoS. His approach is criticized by Gaoue et al. [190]), who note that populations in the studies selected may not associate the human heart with the shape resembling a “Valentine’s heart”, so failure to find a significant proportion of species with cordate leaves being used to treat cardiac disease does not provide a rigorous test of the DoS.

Olaniran et al. [191] surveyed the profiles of sixty plants, belonging to thirty-seven families, which are used for medicinal purposes based on the DoS. They conclude: “This research finds application in future plant exploration and the development of new drugs to combat both ancient and new episodes of human diseases”.

Our literature survey of modern pharmacological studies related to the DoS-specific uses (Table 1) shows that in nine (22.2%) of the cases, there was a positive influence from the plant preparations on these specific DoS uses in laboratory animals. We suggest considering these data as a form of supporting evidence that these DoS uses may also be positive for humans, so that it is not merely an unproven belief.

We thus agree with Durant’s [8] conclusion that whether the DoS can be proved or disproved is a moot point, but it is now well understood that medicinal plant selection is not a random process, and that the DoS has exerted a major influence in identifying the plants we have come to regard as medicinal.

The large endemic plant portion of the endemic DoS uses in present-day Israel and Palestine reflects a deep-rooted local adaptation of a ubiquitous old theory. The incorporation of the adventive species is an indirect form of evidence pointing to an active process of continuing evolution of the DoS in local herbal medicine. The current broad geographic distribution of many of the DoS plant uses (Table 1) may serve as indirect evidence of its current prevalence, and not just as a vestigial manifestation of ancient beliefs.

## 4. Materials and Methods

### 4.1. Study Area and Communities of the Field Study

Our field survey (1999–2005) was an auxiliary part of our study of the sacred trees of northern Israel (details in [192]). The field study (1999–2005) centered on Muslim (Arab, Bedouin) and Druze villages in Galilee. We consider as “Arabs” people who have been settled in their villages for several centuries, “Bedouins,” people who originated from the deserts of Israel and Jordan, migrated to the Galilee during the last three centuries, and were nomadic until the end of the 20th century [193] (p. 30). The Druze are a East Mediterranean group adhering to a religion that was established in Egypt in the 11th century [194] (p. 3). Today, they are concentrated in Lebanon, Syria, and Israel [194] (pp. 8–14).

Data were gathered in 33 villages within the study area. In each village, we made an advance preliminary survey in order to locate those knowledgeable in herbal medicine and popular as medical practitioners; we also approached some people using the snowball method to locate interesting informants. Out of the 118 informants included in this survey, only 27 interviewees (see Appendix A; informants who are cited in the text are indicated in bold numbers) contributed any additional information related to DoS plants. The average age of the informants was 57.7 (+/−14.8) years. The respondents comprised 21 males and six females (in general, women are reluctant to be interviewed, and when they agreed, the interview was held in the presence of other family members). Three of the informants were questioned in our previous survey of the materia medica of Israel [27,28] but were not included in our previous survey [19] due to the small sample size, with less than five informants for each specific DoS use; they are cited here.

### 4.2. Data Collection

Most interviews were performed in Hebrew. If the informant was not Hebrew speaking, the interview was held in Arabic with the help of an experienced interpreter. Due to the refusal of most of the informants to be videotaped or recorded, the whole study is based on oral interviews and field notes that were taken on the spot. Verbal informed consent was obtained from our informants before the interviews. At the end of each interview, we read our notes in the informant’s presence to verify the validity of the data gathered. Most of the people interviewed were active as herbal healers. It is noteworthy that all the informants were illiterate; these people acquired their practical knowledge via active apprenticeship with other experienced healers.

To confirm the DoS uses of the included species, we presented our informants with fresh plants or pictures of the plants that were designated in advance as DoS plants based on our previous study [19]. New additional plant species were incorporated when they were designated as DoS plants by any of our informants. The interviewees were asked “What is the medical use of this plant?” When the answer was suspected by us to be related to the DoS, the next question was, “Why is this plant used for this specific purpose?” Most information concerning the similarity of the plant to a body part or disease symptom was collected via this question. When the interviewee was unable to give a direct answer related to the DoS, we used an indirect approach: We showed him 10 pictures of different plant species, half of which were related to the DoS, and we asked for the medicinal uses of these plants. Additional questions at the end of the interview, indirectly related to the DoS, were: “Which plants are used to treat jaundice, kidney stones, and for a scorpion sting?” By these two complementary approaches, we ensured that the answers were related to the DoS. At the end of the interview, informants were asked if they were aware of the DoS. No one had any previous knowledge of the theory.

In our survey of the literature, we identified plants whose use could be interpreted as examples of the DoS. In most cases in the literature, the original authors do not mention it explicitly (Table 1); e.g., yellow flowers or plants with yellow sap to treat jaundice. This idea fits with the conception of medicinal plants, e.g., in Turkey: “There is an important level of consciousness among the Turks in the use of medicinal plants. In jaundice they use yellow flowering plants, yellow flowers, and yellow roots” [195] (p. 380). We applied this approach in analyzing the cited literature and in other cases such as: kidney-shaped seeds for the treatment of kidney stones, and plants with organs resembling testicles used for fertility.

## 5. Conclusions

We found 43 medicinal species for which at least one traditional use within the study area is related to the DoS. The diversity of health conditions connected with plants in relation to this theory is not high, but should not be dismissed. These uses are based on the similarity of the plant or the plant organ to a damaged human organ, to animal shape or behavior, the similarity of the plant color to the color of the disease, or related to the plant habitat or ecological characteristics. The DoS is still being commonly used in Israel and Palestine. Its current prevalence seems to derive from its strong affinity with Muslim traditional medicine, which is still being practiced. Most of the uses related to this doctrine are not exclusive to our study area, but about one quarter of them are endemic to Israel/Palestine, reflecting local adaptations of the doctrine. We even found uses related to the doctrine for two adventive species, and in our opinion, this serves as evidence that the DoS practice is not necessarily based solely on its traditional historical background but is still evolving locally in accordance with changes in the local flora. The current broad geographic distribution of many of the doctrine’s uses may serve as indirect evidence of its current prevalence and not just as a vestigial presentation of ancient beliefs.

## Figures and Tables

**Table 1:** List of the DoS uses of the Israeli/Palestinian flora, spatiotemporal distribution, and modern pharmacological studies (which are related to the DoS uses).

DoS Reason/Use	Species	Israel/Palestine Author	Other Territories	Modern Pharm. Studies
The seeds resemble kidneys. For kidney stones/Kidney problems	*Alhagi graecorum* Boiss. (=*A. maurorum* Medik.)	[28] (p. 57), [29] (p. 299), [30] (p. 77), [31] (p. 224), [32] (p. 253, Gaza)	**Bahrain**: [33] (p. 2, 5).	[34]
			**Egypt**: [35] (p. 146).	
			**Iran**: [36].	
			**Jordan**: [37] (p. 66).	
			**Saudi Arabia**: [38] (p. 133).	
	*Prosopis farcta* (Banks & Sol.) J.F.Macbr.	[28] (p. 88), [32] (p. 260), [39] (p. 267)	**Egypt**: [40] (p. 337).	
			**Jordan**: [41] (p. 563).	
	*Astragalus macrocarpus* DC.	[19] (p. 332), [29] (p. 308), [42] (p. 330)		
	*Lupinus pilosus* L. (=*L. varius* L.)	[32] (p. 258)	**Jordan**: *L. albus* L. [43] (p. 35).	
	*Coronilla scorpioides* (L.) W.D.J.Koch	[39] (p. 298)		
	*Glycyrrhiza glabra* L.	[29] (p. 299), [39] (p. 298), [44] (p. 8)	**Great Syria**: 13th c., Ibn al-Baytar [45] (III p. 860).	[46]
			**Greece**: [47] (p. 93).	
			**Iraq**: *Kurdish region* [48] (p. 494).	
			**Jordan**: [49] (p. 104).	
			**Morocco**: [50] (p. 93).	
			**Saudi Arabia**: [51] (p. 772).	
			**Turkey**: [52] (p. 71); [53] (p. 106).	
	*Trigonella foenum-graecum* L.	[30] (p. 45), [39] (p. 298), [54] (p. 283)	**India**: [55] (p. 1361).	[56] (p. 921)
			**Iran**: 10th c., [57] (p. 3418).	
			**Iraq**: [58] (p. 813).	
			**Jordan**: [59] (p. 59), [60] (p. 195).	
			**Morocco**: [61] (p. 8).	
			**Saudí Arabia**: [62] (p. 176).	
	*Vicia hybrida* L.	[39] (p. 298)		
	*Cynoglossum creticum* Mill.	[19] (p. 332)		
The plant’s fronds resemble hair/ Hair problems	*Adiantum capillus-veneris* L.	[19] (p. 330)	**Ancient Greece**: 4th–3rd c. BCE: Theophrastus [63] (7.14.10).	[64] (pp. 108–190), [65]
			**Europe**: [66] (p. 6).	
			**India**: [67] (p. 138).	
			**Italy**: [68] (p. 337).	
			**Iran**: [69] (p. 138, *hair color*).	
			**Iraq**: [64] (p. 106).	
			**Jordan**: [41] (p. 553).	
			**Lybia**: [70] (p. 16).	
			**Turkey**: [71] (p. 173).	
The leaves are heartshaped/Heart problems	*Melissa officinalis* L.	[19] (p. 331)	**Cyprus**: [72] (p. 195).	[73,74]
			**England**: [75] (p. 29).	
			**Great Syria:** 13th c., Ibn al-Baytar [45] (I, pp. 73–74).	
			**Greece**: [76] (p. 288), [47] (p. 10).	
			**Iran**: 10–11th c., Ibn Sina [77] (p. 28), [73] (p. 378).	
			**Lebanon**: [78] (p. 146).	
			**Macedonia**: [79] (p. 2064).	
			**Morocco**: [80] (p. 1).	
			**Portugal**: [81] (p. 275).	
			**Serbia**: [82] (p. 98).	
			**Spain**: [83] (p. 214).	
			**Turkey**: [84] (p. 145).	
The fruit resembles a testicle/Fertility, aphrodisiac	*Astragalus macrocarpus* DC.	[19] (p. 330), [32] (p. 254), [42] (p. 230)		
The root has an anthropomorphic shape/Fertility, aphrodisiac, love potion, sexual stimulant	*Mandragora autumnalis* Bertol.	[85] (pp. 115–120)	**Ancient Greece**: 4th–3rd c. BCE: Theophrastus [63] (9.9.1).	
			**Ancient Iran**: 3rd c. CE [86] (p. 58), [87] (p. 378).	
			**Ancient Rome**: 1st c. CE: Dioscorides, [6] (75.10).	
			**Armenia**: [88] (p. 92).	
			**Egypt**: Mamluk Cairo, 12th–15th c. [89] (pp. 167, 168).	
			**Italy**: 16th–19th c. [90] (IV p. 780), [91] (p. 557), [92] (p. 431).	
			**Lebanon**: [93] (p. 259).	
			**Morocco**: [94] (p. 302).	
			**Spain**: [95] (p. 173).	
			**Turkey**: [96] (p. 2), [97] (p. 2).	
The corm resembles a testicle/Fertility, aphrodisiac	*Orchis* spp. (Also: *Ophrys* spp., *Serapias* spp. and related genera)	(**3**, **8**, **13**, **19**, **23**)	**Ancient Rome**: Dioscorides [6] (3.126), Pliny [98] (26.63.97-99, under Satyrion = Orchis sp?).	[99] (*Orhis anatolica* Boiss.)
			**England**: 11–15th c. [100] (p. 230).	
			**Iran**: 10–11th c. Ibn Sina [77] (p. 186).	
			**Iraq**: [101] (p. 70, *Orchis mascula* (L.) L.).	
			**Lebanon**: [99] (*Orchis anatolica* Boiss.).	
			**Turkey**: [102] (*Orchis*, *Ohrys*, *Serapias* etc.,).	
The corm resembles a testicle/Fertility, aphrodisiac	*Colchicum tunicatum*Feinbrun	[103] (p. 73), [104] (p. 168)		
The inflorescence resembles the human male organ	*Cynomorium coccineum* L.	[104] (p. 107), [105] (p. 2)	**Libya**: [70] (p. 17).	[106]
			**North Africa**: [107] (p. 80).	
			**Qatar**: [108] (p. 110).	
			**Saudi Arabia**: [109] (p. 2506).	
The inflorescence is scorpion-like/Scorpion sting	*Heliotropiumeuropaeum* L.	[19] (p. 330)	**Ancient Rome**: Dioscorides [6] (4.190.1).	
			**Cyprus**: [110] (II, p. 55, in the past)	
			**India**: [111] (p. 171).	
			**Iran**: [112] (p. 1).	
			**Oman**: [113] (p. 106).	
			**Pakistan**: [114] (p. 69).	
			**Spain**: [115] (p. 65).	
			**Turkey**: [116] (p. 71), [117] (p. 15).	
	*Heliotropium arabinense* Fresen.	[104] (p. 107)		
Animal behavior resembles human behavior	*Bryza maxima* L.	[19] (p. 330), [85] (pp. 112–113), [118] (p. 127)		
Yellow flowers, yellow decoction/ Treatment of jaundice	*Citrullus colocynthis* (L.) Schrad.	[19] (p. 330), [30] (p. 39); (**4**–**7**)	**India**: [119] (p. 46).	
			**Iraq**: [101] (p. 27).	
			**Jordan**: [41] (p. 559), [37] (p. 67).	
			**Pakistan**: [120] (p. 7).	
			**Qatar**: [121] (p. 263).	
			**Saudi Arabia**: [109] (p. 2505).	
	*Ecballium elaterium* (L.) A.Rich.	[27] (p. 91), [19] (p. 330), [31] (p. 256), [29] (p. 39), [121]; (**4**–**7**)	**Algeria**: [122] (p. 211).	[123] (p. 1124).
			**Ancient Greece:**	
			Dioscorides [6] (4.154).	
			**Ancient Rome**: Galen [124] (XII, p. 122).	
			**Italy**: [125] (p. 23).	
			**Iran**: 10–11th c., Ibn Sina [77] (p. 268).	
			**Jordan**: [126] (p. 925).	
			**Lebanon**: [127] (p. 145).	
			**Lybia**: [70] (p. 17).	
			**North Africa**: [107] (p. 75).	
			**Portugal**: [128] (p. 201).	
			**Turkey**: [53] (p. 106).	
	*Dittrichia* visco*sa* (L.) Greuter (=*Inula* visco*sa* (L.) Aiton)	(**8**–**12**, **25**)	**Italy**: [129] (p. 115).	
	*Glebionis coronarium* (L.) N.N.Tzvel. (*= Chrysanthemum coronarium* L.)	Israel: [85] (p. 149); (**13**–**17**)	**Turkey**: *Glebionis segetum* (L.) Fourr., [71] (p. 124).	
	*Cistanche tubulosa* (Schenk) Hook.f.	[28] (p. 87), [32] (p. 255), [104] (p. 132).	**Saudi Arabia**: [109] (p. 2508).	[130]
	*Calicotome villosa* (Poir.) Link	[131]; (**18**–**20**).	**Palestine**: 6th c., Assaph [132] (IV, p. 396).	
	*Oxalis pes-caprae* L.	(**21**–**24**).	**Lybia**: [70] (p. 19).	
	*Nicotiana glauca* Graham	(**7**, **26**, **27**).	**Jordan**: [133] (p. 138).	
	*Rhamnus alaternus* L.	[28] (p. 41), [29] (p. 301), [42] (p. 254), [134] (p. 44).	**Algeria**: [135] (p. 652).	
			**Iran**: [136] (p. 87).	
			**Lebanon**: [93] (p. 39).	
			**Morocco**: [137] (p. 369).	
			**Sardinia**: [138] (p. 797).	
	*Phillyrea latifolia* L. (=*P.* media L.)	(**9**, **13**, **21**).	**Jordan**: [133] (p. 138).	
	*Pistacia lentiscus* L.	[30] (p. 43), [32] (p. 260), [139]; (**11**, **23**).	**Algeria**: [122] (p. 210).	[133]
			**Arabian Gulf**: [140] (p. 59).	
			**Jordan**: [133].	
			**Oman**: [140] (p. 59).	
	*Nerium oleander* L.	[28] (p. 62), [32] (p. 259).		
	*Asphodelus ramosus* L. (=*A. microcarpus* Salz. & Viv.)	[28] (p. 121), [32] (p. 254), [42] (p. 132), [134] (p. 44).	**Iran**: [136] (p. 87). **North Africa**: [107] (p. 130).	
	*Asphodelus tenuifololius* Cav.	[141] (p. 268), [142] (p. 2117).		
	*Phlomis brachyodon* (Boiss.) Zohary	[143] (p. 268), [142] (p. 2118).	**Jordan**: [126] (p. 924).	
The red fruits resemble blood/Hemorrhage, bleeding	*Rhus coriaria* L.	[143] (p. 396).	**Europe**: [144] (p. 779).	
			**Great Syria**: 13th c. Ibn Al Baytar [45] (III, p. 86).	
			**Iraq**: [101] (p. 81).	
			**Iran**: [145] (Table 1).	
			**Turkey**: [146] (p. 473).	
			**Saudi Arabia**: [51] (p. 772).	
Plant’s roots penetrate rock/ Kidney stones	*Parietaria judaica* L. (=*P. diffusa* Mert &W.D.J. Koch).	[39] (p. 297).	**Jordan**: [147] (p. 300).	
			**Algeria**: [148].	
			**Lybia**: [70] (p. 19).	
			**Malta**: [149] (p. 98).	
			**Spain**: [150] (p. 99), [150] (p. 125).	
	*Phagnalon rupestre* (L.) DC.	[39] (p. 297).	**Jordan**: [147] (p. 300). **Lybia**: [70] (p. 19).	
	*Chiliadenus iphionoides* (Boiss. & Blanche) Brullo (=*Varthemia iphionoides* Boiss.& Blanche)	[39] (p. 297); (**17**, **23**).	**Jordan**: [49] (p. 104), [151] (p. 104), [152] (p. 1).	
	*Ceterach officinarum* DC. (=*Asplenium ceterach* L.).	[39] (p. 296).	**Greece**: [76] (p. 283), [153] (p. 193).	
			**Italy**: [154] (p. 25).	
			**Lebanon**: [93] (p. 136).	
			**Turkey**: [71] (p. 123).	
			**United Arab**	
			**Amirates**: [155] (p. 1314).	
The fronds tremble/Fears	*Adiantum capillus–veneris* L.	[104] (p. 237), [118] (p. 124).		
Leaves like palm of hand/Evil eye	*Ruta chalepensis* L. (=*R. bracteosa* DC.).	[30] (p. 44), [39] (p. 298), [85] (pp. 56–58); (**7**, **14**, **19**, **21**, **23**).	**Ancient Greece**: [156] (p. 471).	
			**Europe**: [157].	
			**Iran**: [158] (*R. graveolens* L.)	
			**Mediterranean**: [156] (p. 471).	
			**Morocco**: [159] (p. 285).	
			**Spain**: [160] (pp. 1165–1167).	
			**Tunisia**: [161] (p. 101).	
			**Turkey**: [162] (p. 103).	
			**Yemen**: [163] (p. 172).	
The plant causes eye inflammation/Eye disorders	*Verbascum sinuatum* L.	[118] (p. 127), [143] (398).	**Morocco**: [94] (302), [50] (p. 95), [164] (p. 475). **North Africa**: [107] (p. 162).	
	*Verbascum eremobium* Murb.	[28] (p. 49).		
The flower’s color resembles an eye inflammation (Lev 2002:17) [165]/Eye disorders	*Glaucium corniculatum* (L.) J.H. Rudolph	[28] (p. 134), [165] (p. 19), [166] (p. 29), [118] (p. 117) (*Glaucium* sp.).	**Great Syria**: 13th c., Ibn al-Baytar [45] (IV, pp. 124–126). **Egypt**: 11–14th c., [167] (p. 286).	
The fist-shaped dry plants open like the palm of a hand when wetted/ Resembles womb’s opening at birth	*Anastatica hierochuntica* L.	[168] (pp. 122–123), [169] (p. 194).	**Bahrain**: [33] (p. 7).	
			**Egypt**: [170] (p. 120).	
			**Iraq**: Kurdish region [48] (p. 403).	
			**Jordan**: [171] (p. 137).	
			**Saudi Arabia**: [38] (p. 131), [172].	
			**Sinai**: [173] (p. 321).	
			**United Arab Amirates**: [155] (p. 1314).	

Bold numbers indicate specific informants according to the list (Appendix A).

## Appendix A. List of the Cited Informants

1. Ḥilwe Batḥīš, 52, Druze, Mas’ade. 21.10.80. (F).
2. Abū Ibrâhīm, 60, Bedouin, Wīdi IMilḥ, 16.6.80. (M.)
3. Abū Qāsem (Ḥasan Abū-ʕālyia), 80, Bedouin, Birğ El-Malikh, 29.6.80. (M.)
4. ʕAli El-ʕAnān (Abū Xāled), 67, Sallāmeh, Bedouin, 2.8.04. (M.)
5. Muṣṭafa Dā’ūd, 56, Šibli, Bedouin, 12.3.05. (M.)
6. Naʽāmeh Muḥammad Ixtilāṭ, 40, Dabūryeh, ʕarab, 3.1.05 (F.)
7. ’Aḥmad ʕaṭa Yāsīn, 70, ʕarab, Ṭamra, 14.6.04. (M.)
8. Šukri ʕāref, 62, Christian, Miʽiliya, 26.6.00. (M.)
9. Saʽīd Maḥmūd Ḥlēiḥel, 80, ʕarab ʕakbara, 28.3.05. (M.)
10. Muḥammed ʕali Fuqara, 58, ʕarab, Buʽeyne Nuğeydāt, 13.11.04. (M.)
11. Šēx Muḥammed Ḥsēn Ilmāz, Bedouin, 90, Kaʽabiyeh, 1.11.11. (M.)
12. Yusuf Nimer Nāṣer (Abū Luṭuf), ʕarab, Saxnīn, 1.1.05. (M.)
13. Ḥammūd Ğawdat, 30, ʕarab, Kabūl, 18.8.00. (M.)
14. Ğamīl Abi Rāed, 71, ʕarab, Mašhad, 29.3.04. (M.)
15. Ḥāmed Abū Muṣṭafa, 62, ʕarrābe, ʕarab, 12.12.03. (M.)
16. Ṭāher Muḥammed, 65, Rummāna, ʕarab, 22.11.04. (M.)
17. ’Aḥmad Abū ʕāmer, 54, Mağd ilKrūm, ʕarab, 28.8.04. (M.)
18. Ğōhara Umm Muḥammed Ḥlēḥel, 80,ʕakbara, ʕarab, 28.3.05. (F.)
19. Maʽarūf ’Aškar, 86, Christian, Kfar Yāsīf, 6.10.04. (M.)
20. ʕādel Abū Ḥāmid, 57, ʕarab, Kafr Manda, 3.6.04. (M.)
21. ’Abū Yiḥya Maḥmūd Ḥmēdi, 97, Bedouin, 1.11.04. (M.)
22. Hādye Sāmiyah, 90, ʕarab, Mazraʽa, 24.8.04. (F.)
23. ’Āmanah Xalīfi, ʕarab, 63, ’Iʽbllīn, 1.12.00 (F.)
24. ʕabd El-Raḥīm Kabha, 60, ʕarab, Umm el-Quṭuf, 26.8.13. (M.)
25. Sleymān Abū Ğušš, 49, Druze, Mghar, 31.12.03. (M.)
26. Tāmir Fallāḥ Mazārīb, 35, Bedouin, Bēt Zarzīr, 11.8.04. (M.)
27. Ruqayya Maghis, 60, Bedouin, Jordēḥ, 11.7.12. (F.)

(M = Male, F = Female).
